# Microbiotoxicity: antibiotic usage and its unintended harm to the microbiome

**DOI:** 10.1097/QCO.0000000000000945

**Published:** 2023-07-25

**Authors:** Anastasia A. Theodosiou, Christine E. Jones, Robert C. Read, Debby Bogaert

**Affiliations:** a Clinical and Experimental Sciences, Faculty of Medicine, University of Southampton, Southampton; b Centre for Inflammation Research, The Queen's Medical Research Institute, University of Edinburgh, Edinburgh, UK

**Keywords:** antimicrobial resistance, antimicrobial stewardship, dysbiosis, microbiome

## Abstract

**Purpose of review:**

Antibiotic use is associated with development of antimicrobial resistance and dysregulation of the microbiome (the overall host microbial community). These changes have in turn been associated with downstream adverse health outcomes. This review analyses recent important publications in a rapidly evolving field, contextualizing the available evidence to assist clinicians weighing the potential risks of antibiotics on a patient's microbiome.

**Recent finding:**

Although the majority of microbiome research is observational, we highlight recent interventional studies probing the associations between antibiotic use, microbiome disruption, and ill-health. These studies include germ-free mouse models, antibiotic challenge in healthy human volunteers, and a phase III study of the world's first approved microbiome-based medicine.

**Summary:**

The growing body of relevant clinical and experimental evidence for antibiotic-mediated microbiome perturbation is concerning, although further causal evidence is required. Within the limits of this evidence, we propose the novel term ‘microbiotoxicity’ to describe the unintended harms of antibiotics on a patient's microbiome. We suggest a framework for prescribers to weigh microbiotoxic effects against the intended benefits of antibiotic use.

## INTRODUCTION

Antibiotics, when appropriately prescribed, are lifesaving, indispensable weapons in our clinical armoury. However, decades of inappropriately broad, lengthy or even unnecessary antibiotics have led to the global emergence of antimicrobial resistance (AMR) [1^▪^]. The WHO deemed AMR one of the 10 greatest threats to global health, and resistant infections are implicated in nearly five million deaths worldwide each year. The spectre of an ’antibiotic apocalypse’ has entered public consciousness, with AMR featuring regularly in the news and social media [2]. The drivers underlying AMR, and the barriers to addressing it, are diverse and complex [3]. Paradoxically, the enormity and pervasiveness of AMR may make it difficult for clinicians to factor into individual prescribing decisions, when faced with the more tangible and immediate problem of the patient in front of them.

In recent years, there has been exponential growth in research into and interest in the human microbiome. Such research has highlighted associations between antimicrobial use, microbiome perturbation, and adverse health outcomes. This review analyses the role of the microbiome as a complex immunological, endocrine and neurological organ system, and the potentially harmful effects of antibiotics on this microbial ecosystem. We propose the novel term ‘microbiotoxicity’ to encompass the unintended side effects of antibiotics on a patient's microbiome. In doing so, we urge clinicians to weigh the benefits of antibiotics in treating infection against these microbiotoxic effects when making individual prescribing decisions. 

**Box 1:**
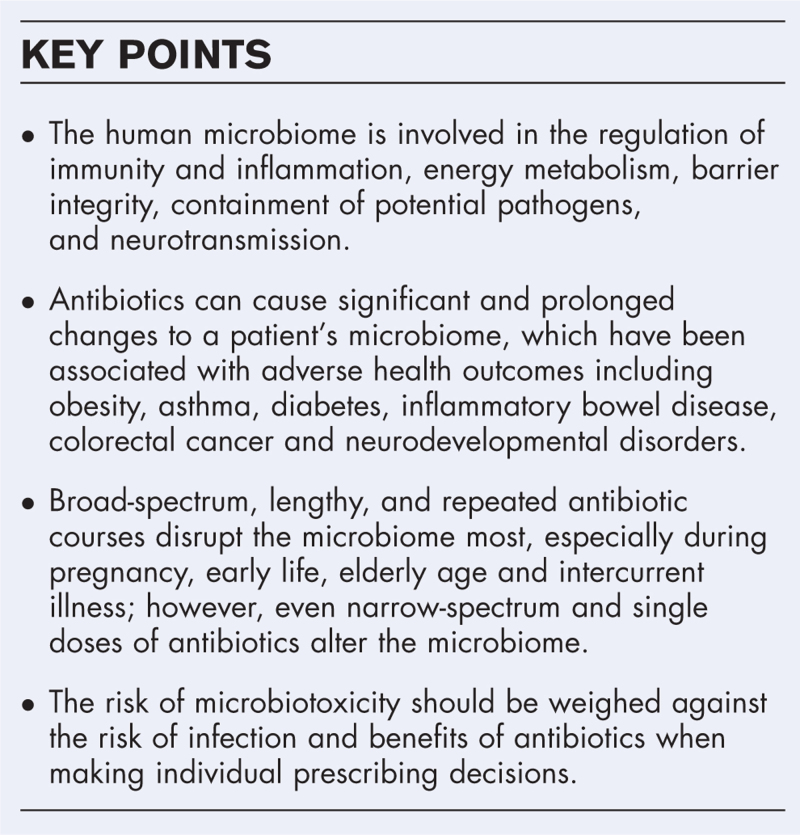
no caption available

## THE MICROBIOME AS A HUMAN ORGAN SYSTEM

**FIGURE 1:**
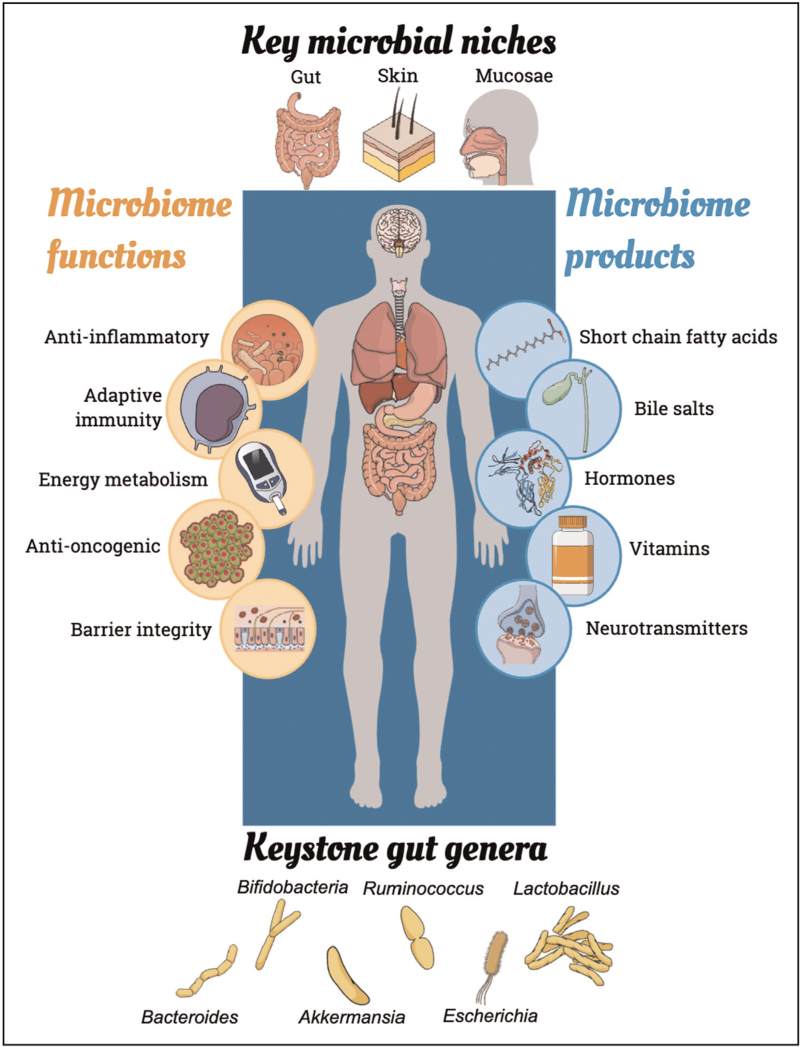
Overview of the microbiome in human health.

The microbiome is the total community of living microorganisms colonizing all outer and inner body surfaces, along with their microbial metabolites, organic compounds and genetic material [4]. There are at least as many bacterial cells as human cells in the body [5], and 150 times more bacterial genes than human genes [6]. The majority of these 30 trillion resident bacteria usually pose no threat to their host; quite the opposite, they are integral to human life (Fig. 1). Many experts now liken the microbiome to an organ in its own right, or even an inextricable component of a human-microbial superorganism called a holobiont [7].

Gut microbes are involved in a wide range of physiological functions, including production of essential vitamins, bile salts and short-chain fatty acids (SCFAs) such as butyrate [8]. These SCFAs suppress oncogenesis, inflammation and appetite; regulate glucose, lipid and energy metabolism; and orchestrate adaptive immunity. Resident microbes are important in the production of neurotransmitters, including dopamine, serotonin and γ-aminobutyric acid, and hormones like glucagon-like peptide 1, and the complex network of neurological, endocrinological and microbial systems involved in homeostasis is termed the ‘gut–microbiota–brain axis’ [9]. Mucosal and skin microbes also play a central role in developing immune tolerance to both microbial and nonmicrobial antigens, and maintaining barrier integrity [10].

Like any organ system, the microbiome demonstrates predictable developmental trajectory. Newborns are born virtually free of bacteria, becoming rapidly colonized with a diverse pioneer microbiome derived largely from their mothers’ vaginal, faecal, skin, mucosal and breastmilk flora [11]. Within days and throughout infancy, the microbiota at each anatomical niche matures until a relatively stable microbiome has been established, with adaptation to environmental and host conditions.

Some argue it is inappropriate to consider the microbiome an organ system, because of its mutability and inter-individual variability [12]. However, while microbiome composition may vary significantly between healthy individuals, the functional and metabolic profiles associated with a healthy microbiome are far more conserved, suggesting a high degree of redundancy [13]. Put another way, there are many ways to construct a healthy microbiome. And there are many ways to harm the microbiome…

## ANTIBIOTICS ARE INHERENTLY MICROBIOTOXIC

**FIGURE 2:**
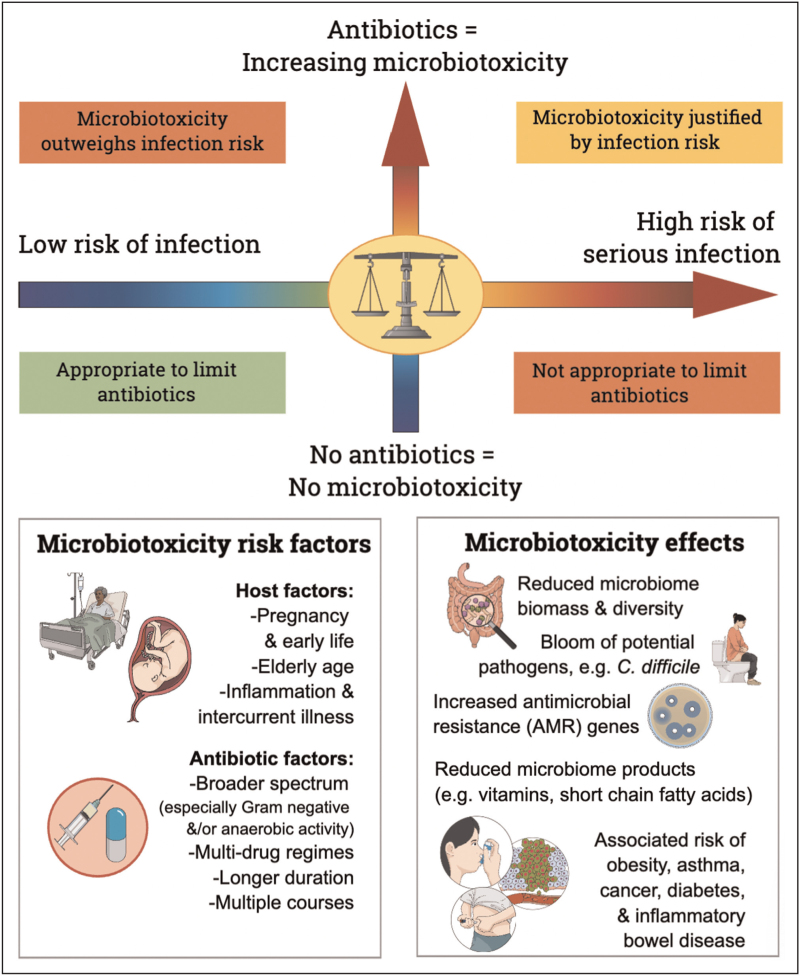
Balancing microbiotoxicity against the need to treat infection.

It might appear redundant to point out that antibiotics kill bacteria; and yet we see no redundancy in warning our patients of the side effects of antibiotics on their own microbiota. We already warn oncology patients of the cytotoxic effects of chemotherapy, and consider liver and renal function tests before prescribing hepatotoxic or nephrotoxic agents. So why not pay heed to our patients’ microbiomes when prescribing microbiotoxic agents (Fig. 2)?

The association between antibiotic use and microbiome perturbation is becoming increasingly compelling, and has been most intensively studied for the gut microbiome [14–16]. Immediately following a course of antibiotics, there is rapid reduction in the total numbers of bacteria (biomass) and bacterial species (alpha-diversity or richness), particularly health-associated keystone bacteria like *Bifidobacterium, Lactobacillus* and *Bacteroides* species [16]. This is accompanied by an initial bloom of potential pathogens that can cause healthcare-associated infections, including Enterobacterales, *Enterococcus*, *Clostridium* and *Candida*[17]. There is also a significant increase in the total burden of AMR genes (the so-called ‘resistome’) in the host's gut following a course of antibiotics [18^▪^,^19^^▪▪^]. This may lead to infections with AMR pathogens, and onward transmission of bacteria carrying AMR genes.

In many cases, antibiotic-associated microbiome perturbations stabilize within a few weeks [15]. However, some studies report much longer recovery time and even incomplete recovery up to a year later [18^▪^,^20^], depending on the type, spectrum, duration and historic use of antibiotics. While the long-term effect of antibiotic-associated microbiome perturbation remains unclear, a growing body of evidence has linked antibiotic-associated microbiome changes with subsequent development of obesity, asthma, diabetes, inflammatory bowel disease and colorectal cancer [21^▪^], as well as neurodevelopmental conditions such as schizophrenia, depression and bipolar disorders [22]. Antibiotic-mediated perturbation is not limited to the gut and has also been demonstrated for the respiratory tract [23] and the vagina [24], with the latter associated with downstream bacterial vaginosis and vulvovaginal candidiasis.

One of the starkest examples of antibiotics driving microbiome dysregulation and remains *Clostridium difficile* diarrhoea, a debilitating acute or chronic infection associated with significant morbidity and cost. Faecal microbiota transplants, which restore host microbiota, are curative in over 80% of treated patients with recurrent *C. difficile* infection, compared with less than one-third of patients treated with vancomycin alone [25], highlighting the importance of overall bacterial communities in keeping pathogens at bay.

It is difficult to estimate the precise morbidity and mortality because of antibiotic-mediated microbiotoxicity. Individual complications such as *C. difficile* diarrhoea have a case fatality rate over 13%, rising to over 26% in elderly patients [26], while 1.27 million deaths worldwide per year are directly attributable to bacterial AMR [1^▪^]. However, the global burden of microbiotoxicity could be much greater indeed, if contributions to noncommunicable diseases such as obesity, cancer and autoimmunity are included [21^▪^].

## HOST FACTORS MATTER

The associations between antibiotic use, microbiome perturbation and ill-health are particularly relevant in early life. Antibiotics in infancy are associated with reduced gut microbiota diversity, AMR gene enrichment and altered longitudinal microbiome evolution relative to untreated infants [27^▪^]. Meta-analyses show that infants receiving antibiotics are 37% more likely to develop asthma than untreated infants, and 82% more likely if antibiotics are given in the first week of life [28^▪▪^]. Significant microbiota changes are also seen in babies whose mothers received peri-partum antibiotics, even if the babies themselves were untreated [29^▪▪^]. The impact of antibiotics on microbiota are also pronounced in the elderly, and in acute inflammation, such as intercurrent infection or comorbidities [14]. And yet, it is our sickest, oldest and youngest patients who receive most antibiotics; indeed, 80% of children aged under 2 years and up to 25% of pregnant women receive at least one course of antibiotics [30]. In a multinational study of over 750 000 full-term and late-preterm neonates, 3% of all newborns received antibiotics for suspected early-onset sepsis; however, for every 58 neonates treated (amounting to 273 antibiotic days), only one case of sepsis was confirmed, suggesting that antibiotic use may have been avoidable in at least some of these neonates [31^▪^]. The effects of antibiotics have also been explored in immunosuppressed patients, such as stem cell transplant recipients, whose immune disturbances and high exposure to antibiotics and healthcare facilities make them particularly prone to antibiotic-mediated dysbiosis [32].

## ANTIBIOTIC CHOICES MATTER

When it comes to your patient's microbiome, some antibiotic choices appear more harmful than others. Antibiotics with broad activity against Gram-negative bacteria, such as ciprofloxacin, are associated with a greater disruption from baseline microbiota than narrower spectrum antibiotics, such as amoxicillin [14]. Broad-spectrum antibiotics and those with activity against health-associated gut anaerobes, including cephalosporins, clindamycin, co-amoxiclav and carbapenems, also carry a greater risk of *C. difficile* infection [33]. Further, combination antibiotics, such as gentamicin with ampicillin, are associated with greater reduction in bacterial richness compared with gentamicin or ampicillin alone [16]. Repeated or longer antibiotic courses also cause greater perturbation, with each additional day of treatment associated with 16–18% reduction in health-associated anaerobes and butyrate-producing bacteria in neonatal ICU patients [34]. That being said, the decision to start antibiotics at all has a greater impact on microbiome disruption than course duration [35]. In fact, microbiota-associated adverse health outcomes have been associated with even a single dose of antibiotics, and with antibiotics not traditionally thought of as high-risk or broad-spectrum, such as macrolides [35].

## ASSOCIATION, CAUSATION AND FUTURE DIRECTIONS

Although most evidence to date is observational, there is interventional data indicating that antibiotics are causally related with downstream microbiome perturbation, including comparisons between different antibiotic regimes [18^▪^]. A prospective trial of 20 healthy volunteers with no clinical indication for antibiotic treatment confirmed that the changes in microbiota diversity and AMR genes are due to antibiotics themselves rather than intercurrent illness [19^▪▪^]. The mechanisms underlying microbiotoxicity are becoming increasingly understood, including direct and indirect effects: bacteria targeted by antibiotics may have co-dependence with other resident bacteria, either producing metabolites required by their symbionts, or degrading waste products toxic to their symbionts [36]. Thus, antibiotics can indirectly harm multiple players in a complex network of symbionts, even beyond their direct spectrum of activity. Antibiotics may also drive ill-health by altering host immune development in early life, including skewing immune development towards T-helper 2-dominant profiles, which may explain the association between antibiotics and downstream allergic sensitisation and autoimmune diseases [21^▪^].

**FIGURE 3:**
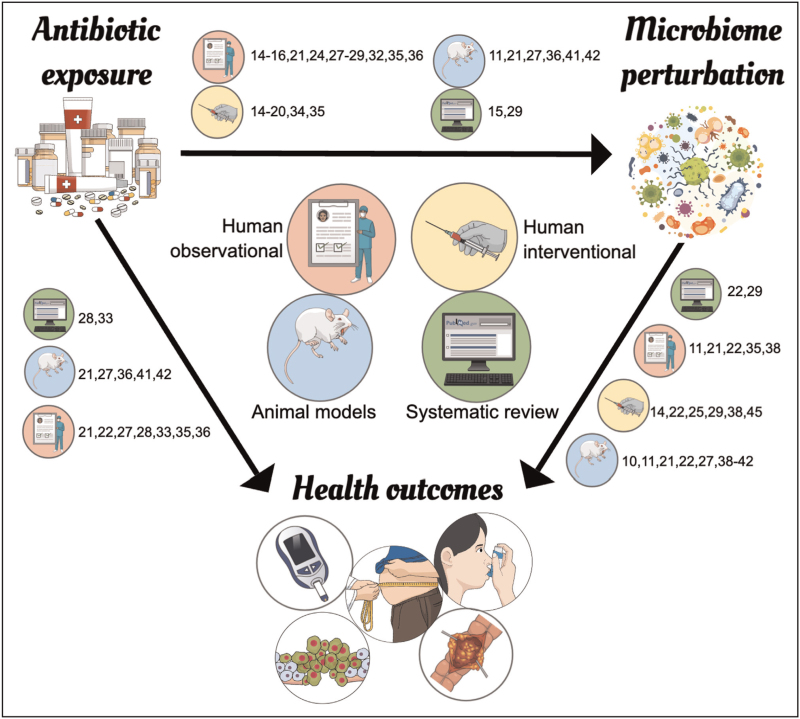
Overview of evidence included: arrows indicate direction of causality implied by evidence. Numbers indicate study cited in reference list.

Such studies, however, do not prove a causal relationship between microbiotoxicity and downstream health outcomes in humans, although several mechanisms have been proposed to explain the associations seen (Fig. 3). In-vitro and animal data suggest that pro-inflammatory bacteria are associated with impaired mucosal barrier integrity and even systemic inflammation [37,38]. A causal role for microbiota dysregulation in disease is also supported by germ-free mouse models. Compared with conventional mice, germ-free mice display profound immune defects and impaired growth [39^▪^]. Moreover, in mouse models for obesity, inflammatory bowel disease and asthma, germ-free mice develop the disease phenotype after receiving stool transplantation from diseased mice, with some evidence of clinical improvement following microbial restoration [40^▪^]. Antibiotics can induce or worsen a pathological phenotype in mice (such as allergic sensitization [41^▪^] or experimentally induced colitis [42]). More compellingly, this phenotype can be recapitulated in germ-free mice receiving stool transplantation from the antibiotic-treated mice [42], or even the offspring of recipient mice [41^▪^]. These findings suggest a causal link between antibiotics and adverse health outcomes, and pinpoint microbiome perturbation (rather than direct antibiotic effects) as the mediator of these downstream phenotypes. However, it remains unclear to what extent findings from germ-free mice can be extrapolated to humans.

As sequencing technologies and bioinformatic analyses continue to improve in efficiency and accessibility, more nuanced research quantifying microbiotoxicity is within reach, and even clinical diagnostic tests based on a patient's microbiome may be on the horizon [43]. Looking ahead, microbiome-based therapies are being investigated to mitigate antibiotic-induced microbial perturbation. RBX2660 (trade name Rebyota) is the first live biotherapeutic product to receive approval (US Food and Drug Administration) for clinical use in recurrent antibiotic-refractory *C. difficile* infection [44]. This consortium of microbes derived from human stool has demonstrated clinical efficacy (treatment success rate 70.6% compared with 57.5% for placebo) in a double-blind randomized placebo-controlled phase III study [45^▪▪^]. However, the real-word utility of such interventions remains unclear.

## CONSIDERING MICROBIOTOXICITY WHEN PRESCRIBING ANTIBIOTICS

We propose the term ‘microbiotoxicity’ when weighing antibiotic side effects on this multitudinous, complex and oft-neglected organ system. By acknowledging the indispensable role of the microbiome in human health, the duty of care of prescribers should be extended to include their patients’ microbiomes. In cases of severe infection, these unfortunate microbiotoxic effects may be entirely justified and unavoidable, and we do not suggest withholding antibiotics when clinically indicated. Rather, each antimicrobial prescription should involve careful weighing of the risk of infection against the risk of antibiotic-induced microbiotoxicity (Fig. 2). Current antimicrobial prescribing guidelines rarely consider these bystander effects on the human microbiome. Although we do not propose ignoring such guidelines, we do recognize that current guidance is necessarily incomplete until microbiome considerations are incorporated. Future strategies for mitigating microbiotoxic effects may include use of probiotics alongside antibiotic courses, with meta-analyses suggesting a role for probiotics in preventing antibiotic-associated diarrhoea [46] and upper respiratory tract infections [47], although further evidence is needed before these can be widely recommended.

The drivers underlying AMR are multifactorial and deeply entrenched, including antibiotic overuse in animal agriculture, population pressure, sanitation and public health infrastructure [3]. Although clinicians may perceive their own patient's clinical needs to be in conflict with tackling the global AMR crisis, invoking the concept of microbiotoxicity may help reframe this by focussing on their own patient's microbial health. This framework may also assist clinicians communicate and negotiate shared decision-making with their patients, especially as public awareness of the microbiome has increased with news and social media reporting [48].

## CONCLUSION

The microbiome is a complex immune, metabolic, endocrine and neurological organ system, integral to the human-microbial superorganism. Antimicrobials are associated with harm to the microbiome and downstream ill-health, although these bystander effects vary with host and antibiotic factors. We hope that the concepts and framework presented here will help clinicians make more nuanced and individualized antimicrobial choices, and even empower them to challenge inappropriate prescribing practices around them. We, therefore, urgently invite our colleagues to add the term ‘microbiotoxicity’ to their clinical vocabulary as a call to arms: a reminder to first do no harm, microbes and all.

## Acknowledgements

*Figures are original and were produced using Mindthegraph.com.*

### Financial support and sponsorship

*AT is funded by a Medical Research Council Clinical Research Training Fellowship (MR/V002015/1).D.B. is supported by a CSO Senior Scottish Clinical Fellowship (SCAF/16/03).*

### Conflicts of interest

*There are no conflicts of interest.*

## REFERENCES AND RECOMMENDED READING

Papers of particular interest, published within the annual period of review, have been highlighted as:

▪ of special interest
▪▪ of outstanding interest
